# Cefazolin as a cause of leukocytoclastic vasculitis

**DOI:** 10.1002/ccr3.992

**Published:** 2017-05-12

**Authors:** Naveed Ali, Nidhi Karia, Richard Goldhahn

**Affiliations:** ^1^ Department of Internal MedicineAbington Memorial Hospital/Abington Jefferson Health1200 Old York RoadAbington19001PA; ^2^ Department of PathologyAbington Memorial Hospital/Abington Jefferson Health1200 Old York RoadAbington19001PA

**Keywords:** Cefazolin, colchicine, corticosteroids, leukocytoclastic vasculitis

## Abstract

Leukocytoclastic vasculitis (LCV) is a cutaneous small vessel vasculitis characterized by cutaneous manifestations in the form of palpable purpura, and rarely bullae, vesicles, and ulcerations. Although rare, cephalosporins such as cefazolin, should be recognized to have a potential to trigger LCV.

## Case Presentation

**Figure 1:**
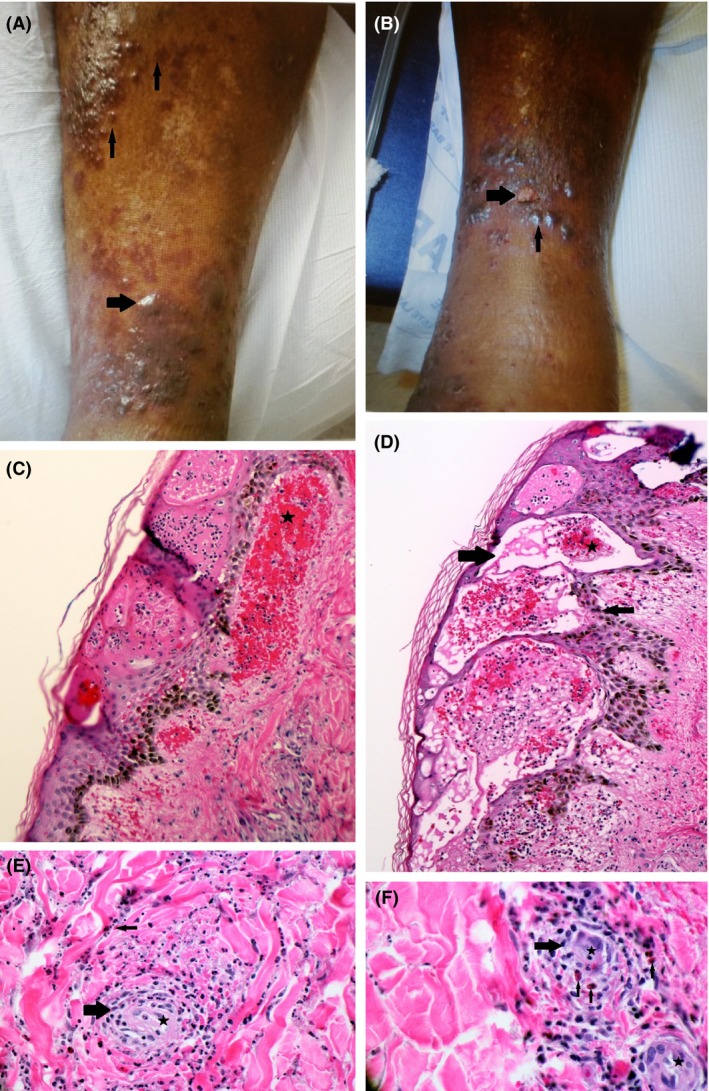
(A) shows palpable purpurae (thin arrows) and an unruptured bullous (thick arrow) on the anterior aspect of the right leg; (B) shows a ruptured bullous (thick arrow) and vesicles (thin arrow) on the anterior aspect of the left leg; (C) shows extravasated red blood cells (star) that give rise to palpable purpura; (D) shows vesicular and spongiotic epidermis (thick arrow), extravasated red blood cells (star) and melanin pigment deposition related to patient's race (thin arrow); (E) shows polymorphonuclear infiltration (thick arrow) around the vessel (star) and areas of nuclear fragmentation or leukocytoclasia (thin arrow); (F) shows polymorphonuclear infiltration (thick arrow) around the vessel (stars) along with eosinophils (thin arrows).

Question 1: What is the diagnosis when a patient develops a rash as shown in Figure 1A and B?

Answer 1: A 67‐year‐old male with a history of hypertension, diabetes mellitus, atrial fibrillation, and right total knee arthroplasty presented with a progressive rash in the lower extremities. Two weeks earlier, he had right knee debridement for septic arthritis caused by methicillin‐sensitive *Staphylococcus aureus* for which cefazolin was initiated for 6 weeks. Upon presentation, his vital signs were within normal limits. Examination demonstrated clusters of palpable, purplish purpurae with associated intact and ruptured blisters scattered over anterior, medial, and lateral aspects of the legs bilaterally below the knees. Laboratory investigations revealed urea of 17 mg/dL, creatinine of 0.89 mg/dL, ESR of 117 mm/h, CRP of 88.14 mg/L, WBC count of 10.6 k/*μ*L without peripheral eosinophilia, hemoglobin of 8.1 g/dL, and platelet count of 312 k/*μ*L. Urinalysis was significant for 2+ proteinuria and microhematuria. Investigations for hepatitis B, hepatitis C, ANA, ANCA, and cryoglobulins were negative. Biopsy of the rash showed perivascular papillary dermal infiltration of polymorphonuclear leukocytes and eosinophils with prominent foci of leukocytoclasis and extravasated RBCs along with a spongiotic intraepidermal and subepidermal bullous change. Based on the clinical presentation, characteristic rash and biopsy results, he was diagnosed with leukocytoclastic vasculitis (LCV) secondary to cefazolin with cutaneous and renal involvement.

Corticosteroids were deferred in light of an ongoing infection and normal renal functions in this patient. Cefazolin was switched to vancomycin and colchicine was commenced with resultant improvement in the rash. When encountered with drug‐induced leukocytoclastic vasculitis, a thorough review of medications should be made to identify the implicated drug and should be discontinued promptly. In the absence of overt organ damage, discontinuation of the offending drug is usually sufficient and immunosuppressive therapy may not be necessary as demonstrated by this case.

Most cases of leukocytoclastic vasculitis are idiopathic; however, drugs, bacterial, or viral infections, sepsis, connective tissue diseases, and underlying malignancies are associated in the rest 1. Histopathologically, the hallmark of diagnosis is perivascular polymorphonuclear infiltration, leukocytoclasia (degranulation and nuclear fragmentation, also known as karyorrhexis) of the neutrophils, and fibrinoid necrosis 2.

## Consent

Written informed consent was obtained from the patient

## Conflict of Interest

None declared.

## Authorship

NA: prepared the entire manuscript. NK, RG: contributed to manuscript preparation.

## References

[1] Martinez‐Taboada, V. M. , R. Blanco , M. Garcia‐Fuentes , and V. Rodriguez‐Valverde . 1997 Clinical features and outcome of 95 patients with hypersensitivity vasculitis. Am. J. Med. 102:186–191. 9217569 10.1016/s0002-9343(96)00405-6

[2] Jennette, J. C. , and R. J. Falk . 1997 Small‐vessel vasculitis. N. Engl. J. Med. 337:1512–1523. 9366584 10.1056/NEJM199711203372106

